# Proceedings of the 2016 MidSouth Computational Biology and Bioinformatics Society (MCBIOS) Conference

**DOI:** 10.1186/s12859-016-1213-4

**Published:** 2016-10-06

**Authors:** Jonathan D. Wren, Inimary Toby, Huxiao Hong, Bindu Nanduri, Rakesh Kaundal, Mikhail G. Dozmorov, Shraddha Thakkar

**Affiliations:** 1 Arthritis and Clinical Immunology Research Program, Oklahoma Medical Research Foundation, 825 N.E. 13th Street, Oklahoma City, OK 73104-5005 USA; 2 Biochemistry and Molecular Biology Department, University of Oklahoma Health Sciences Center, Oklahoma City, USA; 3 Stephenson Cancer Center, University of Oklahoma Health Sciences Center, Oklahoma City, USA; 4 Department of Geriatric Medicine, University of Oklahoma Health Sciences Center, Oklahoma City, USA; 5 Department of Clinical Sciences, UT Southwestern Medical Center, 5323 Harry Hines Boulevard, Dallas, TX 75390-9066 USA; 6 Division of Bioinformatics and Biostatistics, National Center for Toxicological Research, U.S. Food and Drug Administration, Jefferson, AR USA; 7 Department of Basic Sciences, College of Veterinary Medicine, Mississippi State University, Mississippi, MS USA; 8 Bioinformatics Facility, Institute for Integrative Genome Biology, University of California, Riverside, California, USA; 9 Department of Biostatistics, Richmond Academy of Medicine, Virginia Commonwealth University, Virginia, USA

**Keywords:** Bioinformatics, Conferences, MCBIOS, ISCB

## Introduction

The MidSouth Computational Biology and Bioinformatics Society (MCBIOS) held its thirteenth annual conference themed “Precision Medicine and Data Sciences” at the University of Memphis, FedEx Institute of Technology in Memphis, Tennessee on March 3–5, 2016. There were 156 conference registrants and 117 abstracts submitted, including 63 oral and 54 poster presentations.

The conference was co-chaired by Drs. Ramin Homayouni and Shraddha Thakkar. Conference committee members were Cesar M. Compadre (President), Weida Tong (Speaker Coordinator), Darin E. Jones (Treasurer), Bindu Nanduri (Oral talk judge), Mary Yang (Poster judge), Ujwani Nukala (Student Activity Coordinator). For 2017–8, Dr. Bindu Nanduri was chosen as President-Elect and Dr. Shraddha Thakkar as President.

Keynote speakers were: Mary V. Relling, “Clinical Implementation of Pharmacogenetics in Precision Medicine”; Carl E. Cerniglia, “Human Microbiome: Sequencing-Based High-Throughput Omics Technology and Bioinformatics Used in The Assessment of The Safety of Antimicrobial Drug Residues in Food”; Christopher E. Mason, “Genome, Epigenome, Transcriptome, and Epitranscriptome Landscapes: from single cells, to entire cities, to space”; and William Slikker, Jr., “Regulatory Implications of Genomics and Bioinformatics for Food and Drug Safety”.

There were three workshops: Workshop 1: “Using Cyberinfrastructure to Scale your Science” provided by Jason Williams, Ph.D., describing the computational tools and services developed by CyVerse (formerly iPlant Collaborative) to upload, share, and analyze large biological datasets for a variety of applications from genomics to phenotypic analysis. Workshop 2: “Gene Network & Systems Genetics” by Robert Williams, Ph.D., to provide hands on experience with GeneNetwork (www.genenetwork.org). GeneNetwork is one of several web resources that combine public genomic and genetic data along with open-source code for genome-to-phenome analysis. Participants were introduced to human, mouse, rat, and plant genomic datasets and code that can be used in a wide range of bioinformatic, medical, and agronomic settings. Workshop 3: “Next Generation Sequencing Analysis” was provided by Rakesh Kaundal, Ph.D., focused on the analysis of RNA-Seq data for differential gene expression and related statistical tests using R/Bioconductor.

There were 12 breakout sessions. Each session had one featured speaker and four oral presenters presenting their research. Topics were:

1. miRNA and Toxicogenomics
2. Targeted Therapies, Systems Biology and the Future of Safety Assessment
3. Protein Structure/Function and Biological Networks
4. Microbiome: Disease and Drug Resistance
5. Predictive toxicology and Chemo-informatics
6. Next Gen Sequencing
7. Machine Learning in Large Data
8. Proteomics & Host-pathogen interaction
9. Bioinformatics Methodologies
10. Oncology and Precision Medicine
11. Drug Discovery and Development
12. Genomics

The Drug Discovery and Development Colloquium was organized by the student leaders of MCBIOS and was held at the University of Alabama at Birmingham from June 28–30, 2016. The colloquium was organized to showcase the synergistic interaction between chemistry, biology, pharmacology, and bioinformatics in the process of drug development and to promote a professional dialogue between students and experts from different disciplines interested in the processes of drug discovery and development.

**Best Paper Award, MCBIOS 2016**: “VDJML: A file format with tools for capturing the results of inferring immune receptor rearrangements” by Inimary Toby (1st author), Lindsey Cowell (senior author) and 21 co-authors [1].

**Best Oral Presentations (Post-Doctoral fellows):**

- Pankaj Pandey, University of Mississippi
- Aswathy N. Rai, Mississippi State University

**Best Oral Presentations (Students):**

- **First Place**: Renzhi Cao, University of Missouri
- **Second Place**: Chathurani Ranathunge, Mississippi State University
- **Third Place**: Haiou Li, University of Missouri

**Best Poster (Computational Biology):**

- **First Place**: Rachel Steele, Troy University
- **Second Place**: Doga Demirel, University of Central Arkansas
- **Third Place**: Caleb Benson, Mississippi State University, and Vivek Chandramohan, SIT India

**Best Poster (Bioinformatics):**

- **First Place**: Dan Li, University of Arkansas at Little Rock
- **Second Place**: Tanzim Hassan, Mississippi State University
- **Third Place**: Tina Gui, University of Mississippi

## Selecting papers for the MCBIOS XI Proceedings

A total of 27 papers from the work presented at MCBIOS 2016 were submitted to be considered for publication in the Proceedings, and 14 papers were accepted (52 % acceptance rate). At least 2 reviewers anonymously peer-reviewed all submitted papers and acceptable papers were quantitatively ranked on the basis of three evaluation criteria: Novelty (1–5), Impact (1–5) and Clarity (1–3). Editors that were co-authors of submitted papers were not permitted to handle their own papers editorially. Papers generally fell into three categories:

## Networks and pathways

Hyundoo Jeong et al. proposed a probabilistic approach for comparing protein-protein interaction (PPI) networks [2]. The approach estimates the steady-state network flow between nodes of different PPI networks using a Markov random walk model. The proposed approach was evaluated using multiple PPI networks and was found to be accurate and low at computational cost.

Xueyuan Cao et al. describe CC-PROMISE, a method to integrate any two forms of quantitative high-dimensional molecular data such as genotype, copy number, methylation, mRNA expression, miRNA expression, etc., with multiple clinical endpoints for a cohort of patients [3]. This approach identifies genes for which some form of molecular data shows a biologically meaningful association with multiple related end points.

Eshleman and Singh describe an innovative approach to mining social network data from Twitter to extract potential adverse drug events [4]. They look for potential complications along with drug names to identify potentially recurring patterns, leading to the possibility of identifying previously unsuspected adverse events not documented.

Khunlertgit et al. proposed an integrative model to identify subnetworks as molecular markers that relate to cancer status and improve cancer outcome prediction [5]. Such markers can help improve prognosis and diagnosis of cancers, and their study showed that incorporating topological information from prior knowledge to identify the biomarkers may provide additional information for cancer classification.

## Genomics & transcriptomics

Yongsheng Bai et al. developed a program called MMiRNA-Viewer [6] for interactive visualization of the expression relationships between miRNA-mRNA pairs of both tumor and normal samples into a single graph, to help users better explore the relationships between these two entities.

Detection of indels in NGS data has received much less attention than the detection of SNPs. This is in part due to technology limitations, commonly resolved by the “indel realignment” step in bioinformatics pipelines. Vo and Phan described an alternative approach to detect indels by incorporating known genetic variant information in the alignment and variant calling steps [7]. They report improved accuracy in detecting known and novel indels. In particular, their method is well designed to resolve indels that are located in proximity of each other.

Chen et al. address one of the main challenging problems in phylogenetic tree construction, and make a compelling case for the need of improvements in FFP methods [8]. The authors proposed a phylogenetic tree construction method by counting the frequency of triplet translation in prokaryotic DNA, which fully utilized the information contained in genes compared to the traditional FFP-k method, and with lower computational complexity.

In Sujoy Roy et al., the authors encode the co-occurrence of miRNAs and terms within MEDLINE abstracts into a matrix, and then perform singular value decomposition to produce a lower dimensional manifold [9]. They show how the resultant decomposition can be used for miRNA and term queries, and they perform clustering and functional annotation of the miRNAs. The authors present an interesting utilization of unsupervised text mining for the annotation of miRNAs.

Glass and Dozmorov report the impact of parameters such as sample size, cell type-specific proportion variability, mean squared error, etc., on power analysis of linear regression used for estimating cell type-specific gene expression [10]. They incorporate these analyses that can determine the probable significant detection into LRCDE, an R package that performs linear regression cell type-specific differential expression detection on a gene-by-gene basis.

Liu et al. describe a workflow and a database useful for plant geneticists and bioinformaticians [11]. While workflows for sequencing data for human and model organisms have been extensively tested, their application to plant sequencing data is less developed. This workflow is optimized for high-performance processing of plant sequencing data. Extendable to other organisms, it will aid both novices and sequencing data analysis professionals.

## Imaging

Mutlu Mete et al. reported a generalizable machine learning framework and applied the framework in classification of cocaine dependent subjects using brain imaging [12]. This framework reduces data dimension by information theory based feature selection followed by a statistical classifier from machine learning. The results demonstrated the proposed framework is efficient to classify cocaine-dependent and healthy individuals and can be generalized to classifications.

Sertan Kaya et al. describe a novel method for automatic detection of malignancy of the skin lesion in dermoscopy images using the texture homogeneity along the periphery of the lesion [13]. This method performs with high accuracy, and can contribute to early diagnosis of malignant melanoma by distinguishing it from benign lesions.

## Miscellaneous

Inimary Toby et al. describe VDJML, a community standard to annotate V(D)J rearrangements of immune receptors and antibodies [1]. These genes are not germline encoded but rather generated somatically in response to infection. Up until now, the field has lacked a good standard format to report, describe and model sequencing data gathered on these immune receptors. This paper, which won this year’s Best Paper Award, provides a means of representing this data that can be expanded and improved by the immunoinformatics community.

Lee et al. perform a pilot study in Dynamic Topic Modeling (DTM) for the analysis of time-series gene expression data [14]. The authors use DTM to perform unsupervised clustering of time-series gene expression profiles for drug treatment experiments. They map differentially expressed genes as “words” and the drug treatments as “documents” which are then clustered these into “topics”.

## Future meetings

The 14th Annual MCBIOS conference will be held in the Embassy Suites, Little Rock, Arkansas on March 23rd–25th, 2017. The conference theme will be “Bioinformatics and the Development of Therapeutics: Make them Better, Make them Safer”.

## References

[1] Toby IT, Levin MK, Salinas EA, Christley S, Bhattacharya S, Breden F, Buntzman A, Corrie B, Fonner J, Gupta NT, et al. VDJML: a file format with tools for capturing the results of inferring immune receptor rearrangements. BMC Bioinformatics. 2016;17(Suppl 13). doi:10.1186/s12859-016-1214-3. 10.1186/s12859-016-1214-3 PMC5073965 27766961

[2] Jeong H, Qian X, Yoon BJ. Effective comparative analysis of protein-protein interaction networks by measuring the steady-state network flow using a Markov model. BMC Bioinformatics. 2016;17(Suppl 13). doi:10.1186/s12859-016-1215-2. 10.1186/s12859-016-1215-2 PMC5073945 27766938

[3] Cao X, Crews KR, Downing J, Lambda J, Pounds SB. CC-PROMISE effectively integrates two forms of molecular data with multiple biologically related endpoints. BMC Bioinformatics. 2016;17(Suppl 13). doi:10.1186/s12859-016-1217-0. 10.1186/s12859-016-1217-0 PMC5073973 27766934

[4] Eshleman R, Singh R. Leveraging graph topology and semantic context for pharmacovigilance through Twitter streams. BMC Bioinformatics. 2016;17(Suppl 13). doi:10.1186/s12859-016-1220-5. 10.1186/s12859-016-1220-5 PMC5073861 27766937

[5] Khunlertgit N, Yoon BJ. Incorporating topological information for predicting robust cancer subnetwork markers in human protein-protein interaction network. BMC Bioinformatics. 2016;17(Suppl 13). doi:10.1186/s12859-016-1224-1. 10.1186/s12859-016-1224-1 PMC5073942 27766944

[6] Bai Y, Ding L, Baker S, Bai JM, Rath E, Jiang F, Wu J, Jiang H, Stuart G. Dissecting the biological relationship between TCGA miRNA and mRNA sequencing data using MMiRNA-Viewer. BMC Bioinformatics. 2016;17(Suppl 13). doi:10.1186/s12859-016-1219-y. 10.1186/s12859-016-1219-y PMC5073992 27766936

[7] Tran Q, Gao S, Phan V. Analysis of optimal alignments unfolds aligners’ bias in existing variant profiles. BMC Bioinformatics. 2016;17(Suppl 13). doi:10.1186/s12859-016-1216-1. 10.1186/s12859-016-1216-1 PMC5073887 27766935

[8] Chen S, Deng LY, Bowman D, Shiau JH, Wong TY, Madahian B, Lu HH. Phylogenetic tree construction using Trinucleotide Usage Profile (TUP). BMC Bioinformatics. 2016;17(Suppl 13). doi:10.1186/s12859-016-1222-3. 10.1186/s12859-016-1222-3 PMC5073869 27766939

[9] Roy S, Curry BC, Madahian B, Homayouni R. Prioritization, clustering and functional annotation of MicroRNAs using latent semantic indexing of MEDLINE Abstracts. BMC Bioinformatics. 2016;17(Suppl 13). doi:10.1186/s12859-016-1223-2. 10.1186/s12859-016-1223-2 PMC5073981 27766940

[10] Glass ER, Dozmorov MG. Improving sensitivity of linear regression-based cell type-specific differential expression deconvolution with per-gene vs. global significance threshold. BMC Bioinformatics. 2016;17(Suppl 13). doi:10.1186/s12859-016-1226-z. 10.1186/s12859-016-1226-z PMC5073979 27766949

[11] Liu Y, Khan SM, Wang J, Rynge M, Zhang Y, Zeng S, Chen S, JV M, Valliyodan B, Calyam PP, et al. PGen: large-scale genomic variations analysis workflow and browser in SoyKB. BMC Bioinformatics. 2016;17(Suppl 13). doi:10.1186/s12859-016-1227-y. 10.1186/s12859-016-1227-y PMC5074001 27766951

[12] Mete M, Sakoglu U, Spence JS, Devous Sr. MD, Harris TS, Adinoff B. Successful classification of cocaine dependence using brain imaging: a generalizable machine learning approach. BMC Bioinformatics. 2016;17(Suppl 13). doi:10.1186/s12859-016-1218-z. 10.1186/s12859-016-1218-z PMC5073995 27766943

[13] Kaya S, Bayraktar M, Kockara S, Mete M, Halic T, Field HE, Wong HK. Abrupt skin lesion border cutoff measurement for malignancy detection in dermoscopy images. BMC Bioinformatics. 2016;17(Suppl 13). doi:10.1186/s12859-016-1221-4. 10.1186/s12859-016-1221-4 PMC5073935 27766942

[14] Lee M, Liu Z, Huang R, Tong W. Application of dynamic topic models to toxicogenomics data. BMC Bioinformatics. 2016;17(Suppl 13). doi:10.1186/s12859-016-1225-0. 10.1186/s12859-016-1225-0 PMC5073961 27766956

